# Small Molecule, Big Prospects: MicroRNA in Pregnancy and Its Complications

**DOI:** 10.1155/2017/6972732

**Published:** 2017-06-20

**Authors:** Meng Cai, Gopi K. Kolluru, Asif Ahmed

**Affiliations:** Aston Medical Research Institute, Aston Medical School, Aston University, Aston Triangle, Birmingham B4 7ET, UK

## Abstract

MicroRNAs are small, noncoding RNA molecules that regulate target gene expression in the posttranscriptional level. Unlike siRNA, microRNAs are “fine-tuners” rather than “switches” in the regulation of gene expression; thus they play key roles in maintaining tissue homeostasis. The aberrant microRNA expression is implicated in the disease process. To date, numerous studies have demonstrated the regulatory roles of microRNAs in various pathophysiological conditions. In contrast, the study of microRNA in pregnancy and its associated complications, such as preeclampsia (PE), fetal growth restriction (FGR), and preterm labor, is a young field. Over the last decade, the knowledge of pregnancy-related microRNAs has increased and the molecular mechanisms by which microRNAs regulate pregnancy or its associated complications are emerging. In this review, we focus on the recent advances in the research of pregnancy-related microRNAs, especially their function in pregnancy-associated complications and the potential clinical applications. Here microRNAs that associate with pregnancy are classified as placenta-specific, placenta-associated, placenta-derived circulating, and uterine microRNA according to their localization and origin. MicroRNAs offer a great potential for developing diagnostic and therapeutic targets in pregnancy-related disorders.

## 1. Introduction

**Figure 1:**
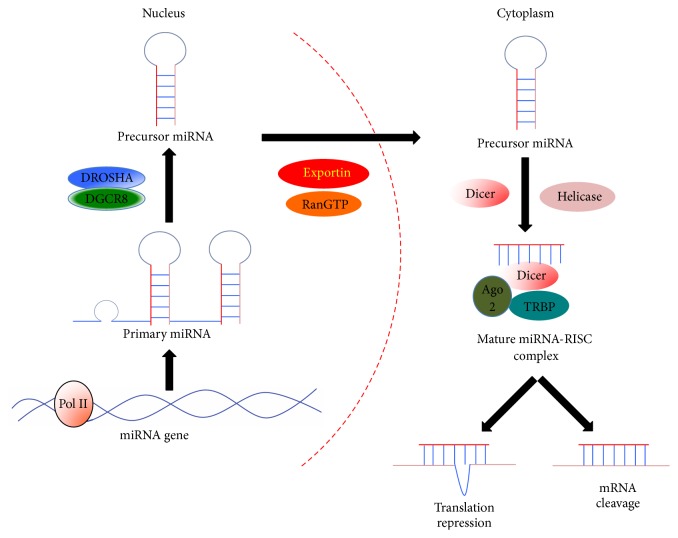
A schematic diagram showing miRNA biogenesis and miRNA-mediated target mRNA suppression. The primary miRNAs (pri-miRNA) are transcribed from miRNA genes by RNA polymerase II or III in the nucleus and subsequently processed by Drosha and DGCR8 to form precursor miRNA (pre-miRNA). Next, the pre-miRNAs are exported into cytoplasm by exportin 5 and RanGTP. In the cytoplasm, the pre-miRNAs are further cleaved by Dicer and resulted in two ssRNAs. Finally, the ssRNAs integrate into RISC protein complex which includes Argonaute 2, Dicer, and TRBP. Functionally, the miRNA-RISC complex inhibits target mRNA expression through either translational repression or mRNA cleavage.

RNA is a single-stranded genetic material involved in various biological roles including coding and decoding, regulation of gene expression, and protein synthesis. Noncoding RNAs (ncRNA) that are not translated into protein form a large portion of total cellular RNA (95–98%). The ncRNAs include some highly abundant RNAs, such as transfer RNA (tRNA) and ribosomal RNA (rRNA), and functionally important RNAs, including long ncRNA and microRNA (miRNA) [1, 2]. Typically, miRNAs are transcribed from miRNA genes by RNA polymerase II or III in the nucleus. The primary transcripts of miRNA genes (pri-miRNA) are single-stranded RNA molecules composing several hundred RNA nucleotides and one or more stem-loop structures. Subsequently, the stem-loop structure is recognized and processed by a protein complex containing ribonuclease Drosha and double-stranded RNA binding protein DGCR8 to form precursor miRNA (pre-miRNA) [3]. Next, the pre-miRNAs are exported into cytoplasm by exportin 5 utilizing the energy provided by Ran- (ras-related nuclear protein-) GTP complex. In the cytoplasm, the loop and part of the stem structure of pre-miRNA are cleaved by Dicer to form a double-stranded RNA (dsRNA) molecule with 19–25 nucleotides [4]. Finally, the dsRNA dissociates into two single-stranded RNAs (ssRNA) with the help of helicase and the ssRNAs subsequently integrate into the RNA-induced silencing complex (RISC), including Dicer, transactivation response RNA binding protein (TRBP), and Argonaute 2 (Ago 2), to target downstream messenger RNA (mRNA) by incomplete alignment [5]. Functionally, the miRNA-RISC inhibits target mRNA expression through either translational repression or mRNA cleavage (Figure 1). Notably, miRNAs in human are estimated to target well over 60% of total coding genes [6]. So far, more than 1000 and at least 303 mature miRNAs were discovered from human and mouse genomes, respectively [7, 8]. Given the broad range of miRNA targets in the cell, it is no surprise that miRNAs regulate many physiological and pathological processes [9–11].

**Figure 2:**
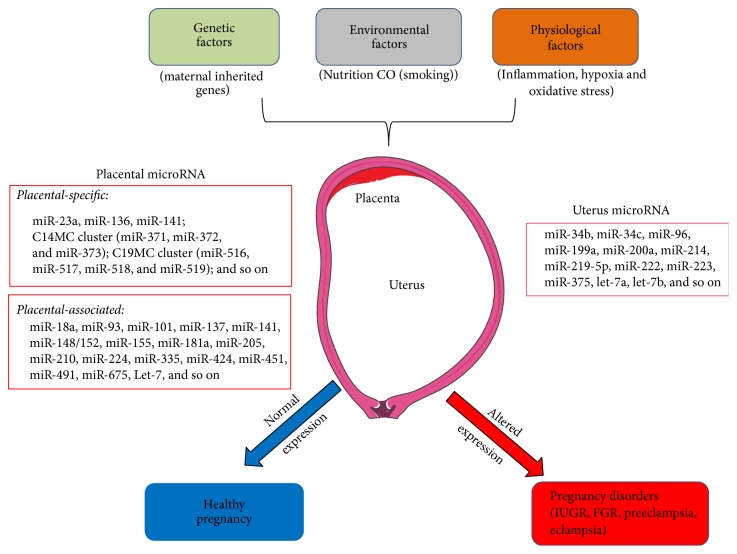
The pregnancy process is regulated by genetic, environmental, and physiological factors. MiRNAs in the placenta and uterus respond to the change of these factors during pregnancy. Altered expression of miRNAs leads to the pregnancy disorders.

Placenta and uterus are the major organs in pregnancy. Of the two, placenta is the most important one and is associated with many pregnancy-related disorders. Placenta facilitates nutrient uptake and gaseous exchange to the developing fetus. It regulates temperature, produces hormones, and provides protection from internal infections during pregnancy. Placenta is mainly composed of trophoblasts, decidual cells, endothelial cells, and mesenchymal cells. The cellular activities of these cells, such as trophoblast proliferation, differentiation, and invasion, as well as mesenchymal cell differentiation, decidualization, and angiogenesis, are pivotal for a healthy pregnancy [12, 13]. Moreover, pregnancy is an altered physiological condition regulated by genetic (e.g., maternal inherited genes), environmental (e.g., nutrition), and physiological (e.g., inflammation, hypoxia) factors. Many studies suggest that miRNA expression is altered due to the response to these changes [14–16]. Thus, we propose that miRNAs respond to the change of physiological condition during pregnancy and facilitate successful pregnancy process, whereas dysregulation of miRNAs causes or contributes to disorders of pregnancy (Figure 2).

## 2. MicroRNA in Pregnancy Regulation

The importance of miRNA in the regulation of pregnancy has been proved by knocking down Dicer in mouse model or human tissue. Deletion of Dicer caused hypotrophy and disorganization of uterus and oviduct which subsequently resulted in female sterility [17]. Interestingly, global reduction of miRNA caused by the deletion of Dicer in human placental explants enhanced cytotrophoblast proliferation by activating promitogenic signaling pathways [18].

A large number of miRNAs have been identified to be associated with pregnancy and its disorders using quantitative PCR, microarray, or deep sequencing profiling [19–21]. These miRNAs can be classified as tissue-resident and circulating miRNA, based on their physiological localization and transportation method. The tissue-resident miRNAs locate in the tissue where they are being synthesized and regulate target genes locally, whereas circulating microRNAs are released into the blood via exosome and transported to other cell/tissue types to regulate target genes in a paracrine or telecrine action [22]. In pregnancy, since the placenta and uterus are the main pregnancy-related organs, tissue-resident miRNAs discussed in this review include placental (placenta-specific and associated) as well as a small group of uterine miRNAs (Table 1). This review will mainly focus on the recent progress of miRNAs in pregnancy-related complications, such as pregnancy loss, preeclampsia, intrauterine growth restriction/fetal growth restriction (IUGR/FGR), and preterm birth. Circulating miRNAs released from placenta will be discussed briefly due to limited literature. Moreover, the fetomaternal miRNA exchange and its role in the pregnancy regulation will also be mentioned.

## 3. Placental MicroRNA

There seems to be a confusion in the literature on the terminology and we will clarify it here. Typically, placental miRNAs include placenta-specific, placenta-associated, and placenta-derived circulating miRNAs. While placenta-specific miRNAs are expressed largely or uniquely in the placental tissue, placenta-associated miRNAs are expressed ubiquitously in the placenta and other tissues. Placenta-derived circulating miRNAs refer to the placenta-released circulating miRNA. Considering that placenta is a central organ for healthy pregnancy, studies regarding the placental miRNAs are important to understand the regulatory mechanisms of normal and complicated pregnancies [96].

### 3.1. Placenta-Specific MicroRNA

The observation of placenta-enriched miRNAs, miR-141, miR-23a, and miR-136, was first reported in 2004 [23]. Benefitting from a large-scale small RNA library sequencing, placenta-specific/enriched miRNAs have been proved to be abundant (~100) in the mammalian genome [7, 29]. Interestingly, many of these placenta-specific miRNAs are closely located on their respective chromosomes as a cluster and are regulated by the same promoter. C19MC, a cluster on chromosome 19, was first identified in 2009 [97], followed by C14MC, a cluster on chromosome 14, and miR-371-3 cluster, also on chromosome 19 [26]. Expression of C19MC members is largely restricted in the reproductive system and placenta [29, 30]. Similarly, C14MC accommodates 52 miRNA genes in about 40 kb region and the expression of members is abundant in developing embryo and placental tissue [26], whereas miR-371-3 cluster only has 3 main members and is prominently expressed in the placenta [33]. Surprisingly, some placenta-specific miRNA clusters can be located even within a single gene intron. The* Sfmbt2* cluster, which is located in the 10th intron of* Sfmbt2* gene, has 36 distinct miRNAs which are abundantly expressed in the embryonic stem cells and placenta [98]. In addition, some placenta-specific miRNAs, located in different chromosomes, are derived from the same transposon. For example, miR-1302 family, which comprises 58 orthologs, is derived from MER53 transposon and is only found in the mammalian placenta [35].

#### 3.1.1. Placenta-Specific MicroRNA Regulates Pregnancy Process

Placenta-specific miRNAs have long been proposed for their possible regulatory role in normal and complicated pregnancies. Until now, studies of placenta-specific miRNAs in the regulation of pregnancy are very limited focusing only on the basic biological characteristics. Using high-throughput profiling array and quantitative PCR, abundant expression of C19MC members was located in the primary human trophoblasts [83]. Moreover, cluster members of C19MC were also shown to be abundantly expressed in the placenta-derived mesenchymal stromal cells (PDMSC), suggesting a potential regulatory role in the stem/progenitor cells [99]. Furthermore, the C19MC members are shown to be temporally expressed. For instance, placenta-specific miRNAs, including miR-141 and C19MC family, were differentially expressed in various development steps to meet the different regulatory demands of pregnancy [100, 101].

Recently, the implication and possible regulation of these miRNAs in normal and complicated pregnancy were revealed partly. It was demonstrated that placenta-specific miRNAs, miR-141 and miR-519d-3p (a member of C19MC), regulate trophoblast cell proliferation, invasion, migration, and intercellular communication [24, 27, 32]. Furthermore, Keniry and colleagues demonstrated that overexpression of placenta-exclusive miR-675 inhibited embryonic and extraembryonic cell proliferation [34]. Notably, underexpression of four C19MC members, miR-517a, miR-517b, miR-518b, and miR-519a, was observed in complete hydatidiform moles (CHM) [31]. Likewise, seven members of C19MC cluster, miR-518b, miR-1323, miR-516b, miR-515-5p, miR-520h, miR-519d, and miR-526b, were significantly downregulated in the placenta of FGR patients and four of them (miR-518b, miR-1323, miR-520h, and miR-519d) were confirmed as FGR-associated placenta-specific miRNA [28]. Furthermore, placenta-specific miR-141 was identified to target pleiomorphic adenoma gene 1 (PLAG1), an important regulator of insulin-like growth factor 1 (IGF-1) that contributes to FGR [25].

### 3.2. Placenta-Associated MicroRNA

Placenta-associated miRNAs are expressed ubiquitously in placenta and other tissues. Like placenta-specific miRNAs, the placenta-associated miRNAs show different expression profiles in various gestational ages of trophoblast cells, placental tissue, and maternal plasma. A total of 45 miRNAs were identified to be differentially expressed in the trophoblast cells of first- and third-trimester placentas with 58% of these microRNAs being placenta-associated [100]. Recent study on miRNA profiles in human placenta identified 191 differentially expressed microRNAs between first and third-trimester placentas, including both placenta-specific and placenta-associated microRNAs [102]. More importantly, this study revealed that oncogenic, angiogenic, and antiapoptotic miRNAs were dominantly expressed in the first-trimester placentas, whereas expression of miRNAs related to cell differentiation and tumor suppression was predominant in the third-trimester placentas. Furthermore, in the maternal plasma, a substantial amount of placental-associated miRNAs was differently expressed during the first-, second-, and third-trimester gestation [103].

#### 3.2.1. Placenta-Associated MicroRNAs Response to Altered Inflammation and Hypoxia Condition in Pregnancy

Different expression of miRNAs in the placenta during gestation depends on regulatory demand of the physiological change, such as inflammation and hypoxia [104, 105]. Let-7 functions downstream of NF-*κ*B signaling pathway and negatively regulates IL-6 expression. Strong expression of let-7 was detected in the placenta and amnion, implying the possible regulation on placental inflammation [36]. Similarly, miR-181a blocks the activation of TGF-*β* signaling pathway and enhances expression of IL-6; thus increased expression of miR-181a in the placenta attenuates the immunosuppressive properties of mesenchymal stem cells and contributes to the abnormal pregnancy [63]. On the other hand, the miR-148/152 family negatively regulates innate immune responses, mediating immune tolerance to facilitate a healthy pregnancy [56, 57].

Since the placenta is relatively hypoxic in the early stage and pathological condition of pregnancy [106, 107], placenta-associated miRNAs also respond to this hypoxic challenge. Interestingly, although hypoxia treatment did not affect the global miRNA biosynthetic pathway in primary trophoblast, the individual miRNA exhibited different expression patterns under hypoxia [108]. A set of seven placenta-associated miRNAs, miR-93, miR-205, miR-224, miR-335, miR-424, miR-451, and miR-491, were differentially expressed in primary trophoblasts exposed to hypoxia and miR-205 was confirmed to target an important placental development factor, MED1 [51]. In cigarette smoke-exposed placenta, miR-16, miR-21, and miR-146a were significantly downregulated [39]. Further studies confirmed that placenta-associated miRNAs regulate mitochondrial electron transport and renin-angiotensin system adaptation to hypoxia. HIF-responsive miR-210 was increased in hypoxic placenta which compromised mitochondrial electron transport chain function and energy metabolism [69]. In antenatal maternal hypoxia (AMH), the expression of placenta-associated miRNAs, miR-199b, miR-27a, and miR-429, was reduced to allow an increase of some important renin-angiotensin system factors that help placental adaptation to hypoxia [47].

#### 3.2.2. Placenta-Associated MicroRNAs Regulate Pregnancy-Associated Cellular Activities

Many placental miRNAs were identified as the regulators of pregnancy-related trophoblasts and endothelium cellular activities. Members of the miR-17~92 cluster and its paralogs, miR-106a~363 and miR-106b~25, were significantly downregulated to facilitate syncytiotrophoblast differentiation [41]. Knockdown of global miRNA synthesis and individual miRNA, such as miR-675, enhanced trophoblast proliferation [18, 81]. Reversely, some miRNAs were upregulated to enable trophoblast proliferation, migration, and invasion. For example, overexpression of miR-378a-5p and miR-376c enhanced these processes [77, 78]. So far, only a handful of miRNAs, including miR-424 [79], miR-101 [52], miR-18a [46], miR-335 [50], miR-137 [55], and miR-155 [61], have been implicated in trophoblast dysfunction through various targets, including endothelial nitric oxide synthase (eNOS) and soluble vascular endothelial growth factor receptor 1 (sVEGFR-1 or sFlt-1).

Angiogenesis is another critical physiological activity in the placenta during early pregnancy [13]. MiRNA profiles in porcine placenta of different gestational age revealed altered expression of some angiogenic-associated miRNAs, such as miR-92, miR-17, and miR-27 [42]. In preeclampsia patients, miR-126, a proangiogenic factor which correlates with VEGF expression, was decreased [53]. On the contrary, miR-15b, which negatively regulates angiogenesis in endothelial cells, was shown to be increased in preeclamptic placentas [38].

#### 3.2.3. Placenta-Associated MicroRNA in Pregnancy Complications

Since placenta-associated miRNAs are important pregnancy regulators, the aberrant expression of these miRNAs is associated with various disorders of pregnancy. Microarray profiling and further quantitative PCR analysis revealed that miRNAs are differentially expressed in the placenta and circulation of preeclamptic women. The first study was published in 2007 where the group identified two differentially expressed miRNAs between preeclamptic and normal placentas [109]. Two years later, two studies using a microarray approach identified seven and thirty-four differentially expressed miRNAs in preeclamptic placentas, respectively [65, 67]. Recently, more placenta-associated miRNAs have been found to be dysregulated in preeclamptic placental tissue [110–112]. However, results from these studies are elusive, even though similar approaches have been employed. Due to the complexity of placenta composition and the different gestational age upon sample collection, identified miRNA profiles can vary or present completely opposite results between studies, for example, miR-195 [65–67]. Other studies, using next generation sequencing, determined different miRNA expression profiles in the circulation of preeclampsia and healthy pregnant women. Dysregulated expression of over 29 miRNAs was observed in the plasma or serum of preeclamptic placentas, including both placenta-specific and placenta-associated circulating miRNAs [113, 114]. Like the placental studies, these results are confusing and conflicting, but, on the other hand, they also confirmed the implication of miRNAs in the pathogenesis of preeclampsia and provided potential miRNA candidates for further research.

#### 3.2.4. miR-155 and miR-210 in the Pathogenesis of Pregnancy Complications

Currently, only a handful placenta-associated miRNAs have been characterized for their function and downstream targets in preeclampsia. Among these, miR-155 and miR-210 are the most extensively examined ones. Using preeclamptic placental tissue, the first study revealed that miR-155 targeted and downregulated angiogenic factors, thus contributing to the pathogenesis of preeclampsia [62]. Cheng and colleagues found that miR-155 was downregulated in the preeclamptic patient endothelial cells. Further study identified another critical preeclamptic pathogenic factor, angiotensin II type 1 receptor, as a target of miR-155 [58]. In addition, miR-155 was linked to the trophoblast function, including proliferation, invasion, and differentiation [59, 60]. Recently, miR-155 was shown to modulate eNOS expression in trophoblast cells [61]. Another highly studied miRNA, miR-210, was first identified in trophoblast cells as an iron metabolism regulator responding to hypoxia stress and implicated in the defective placentation [71]. Later, miR-210 was confirmed to be upregulated in preeclampsia, resulting in negative regulation of trophoblast cell migration and invasion [68, 75]. A recent study further identified its function in the modulation of mitochondrial respiration in placenta which contributes to preeclampsia [74]. Other studies revealed that miR-210 modulates inflammation pathway, potassium channel, and thrombospondin in order to contribute to preeclampsia [70, 72, 73].

#### 3.2.5. Other MicroRNAs in the Pathogenesis of Pregnancy Complications

Apart from these two miRNAs, there are many other miRNAs that are associated with preeclampsia. For instance, miR-325 was elevated in the preeclamptic patients and correlated with the patient blood pressure [76]. Notably, miRNAs related to the dysregulation of trophoblast and stem cell function are also associated with preeclamptic pathophysiology. MiR-378a-5p, miR-195, and miR-29b, which regulate trophoblast cell proliferation, invasion, migration, and angiogenesis, were dysregulated in preeclamptic placentas [48, 64, 78]. Recent study further confirmed the link between mesenchymal stem cells (MSCs) related miRNAs and pregnancy complications. MiRNA expression profile in MSCs derived from serve preeclamptic patients showed significant differences compared to healthy controls [115]. MiR-494 inhibited MSCs proliferation and angiogenesis potential [80], whereas miR-30a attenuated MSCs-mediated immune response [44]. Furthermore, miRNAs that regulate important physiological signaling pathways are also implicated in preeclampsia. For example, angiogenesis-associated miRNAs, miR-17, miR-20a, and miR-20b, together with miR-21 which negatively regulates hydrogen sulfide generation, were significantly increased in preeclamptic placentas [45, 116].

Placenta-associated miRNAs are also implicated in other pregnancy complications, such as small-for-gestational age (SGA), FGR, or IUGR. Although no difference was identified in the miRNA profiles between SGA patients and control, seven miRNAs were identified to be differentially expressed between combined SGA/preeclampsia and control placentas [109]. Similar studies using maternal FGR plasma confirmed that individual miRNA expression in the FGR and control patients exhibited no difference. However, a total 1.8-fold elevation in expression of a selected placenta-specific and placenta-associated miRNA group was noticed [51]. Furthermore, individual miRNAs have been implicated in SGA patients. For example, placental angiogenic-associated miRNAs, miR-16 and miR-21, were markedly decreased in the SGA placentas, suggesting an additive effect in SGA pathogenesis [40].

In addition, placenta-associated miRNAs are implicated in some maternal-related pregnancy disorders, for example, preterm birth and abortion. Mayor-Lynn and colleagues analyzed the miRNA expression profiles between preterm and normal term placentas. They identified twenty differentially expressed miRNAs, including some well-known preeclamptic or SGA-associated miRNAs [37]. In early pregnancy loss, placental expression of miR-17 and 19b was downregulated compared to the matched healthy controls [43]. MiR-133a was shown to be overexpressed in the recurrent spontaneous abortion (RSA) patient villi that led to the downregulation of HLA-G [54]. Moreover, in the placenta accreta patients, miR-34a expression was decreased in trophoblast cells and the suppression of miR-34a increased trophoblast invasive potential [49].

### 3.3. Placental-Derived Circulating MicroRNA

Since miRNAs are known to be released into circulation [22], efforts have been made to identify the expression profiles of these miRNAs in maternal circulation and explore their diagnostic potential [66, 113].

The placental-specific C19MC members were first discovered in the maternal blood and proved to be released from the trophoblasts via exosomes [83]. Upregulation of circulating C19MC miRNAs was identified as a consequence of preeclampsia onset [85]. Furthermore, Kotlabova and colleagues tested the expression of some known placental-specific miRNAs and found seven members of C19MC cluster in the plasma [84]. In a follow-up study, expression of these seven microRNAs was increased in the circulation of women who were at early gestational age and later developed pregnancy disorder [117]. Interestingly, work from the same group demonstrated the downregulation of some C19MC miRNAs in the placenta of pregnancy-related complications [118]. In a recent study, profile of circulating placental-specific miRNAs has been comprehensively identified [82]. Notably, circulating placenta-specific miRNA clusters, miR-498 (46 individual microRNAs), miR-127 (8 individual microRNAs), and miR-134 (41 individual microRNAs), fell within the range of C19MC and C14MC clusters. Furthermore, this study identified unique expression pattern of miRNA clusters in the maternal and fetal circulation, but not in the circulation of men or nonpregnant women. Interestingly, the miRNA fingerprints/profiles between primary isolated trophoblast cells and trophoblast cell lines display major differences, implying possible miRNA transportation and exchange from other cell types [100].

#### 3.3.1. Placenta-Derived Circulating MicroRNA as Biomarker

Placenta-derived circulating miRNAs were initially introduced as biomarkers for pregnancy monitoring. The miRNA expression profiles in maternal plasma before and after parturition were analyzed and differently expressed miRNAs, including many placental-associated miRNAs, were further confirmed by qPCR [119]. Interestingly, four of the most abundant placental miRNAs, miR-141, miR-149, miR-299-5p, and miR-135b, were also detectable in the maternal plasma during pregnancy and followed by decrease after delivery [86]. To confirm the biomarker potential, expression profiles of these circulating miRNAs in the blood of pregnant and nonpregnant women, under different pathophysiological conditions, have been studied. In the serum of pregnant women, miRNAs associated with placenta were significantly elevated compared to nonpregnant women and their expression levels correlated with pregnancy stages [120]. Moreover, a group of selected hypoxia-regulated and placenta-specific miRNAs elevated 1.84-fold in the plasma of women with FGR [51]. Importantly, the expression of these selected miRNAs in the FGR women placenta was reduced when compared to normal pregnant women, suggesting the reverse correlation of miRNA expression in the circulation and placenta. However, the diagnostic potential of placental-derived miRNA is still debatable with the recent evidence that the expression of four members of C19MC did not show significant difference between FGR and normal pregnancy [28]. It seems that a miRNA pool rather than individual miRNAs has a defined role as a biomarker. In addition, maternal circulating miRNAs can be used as potential biomarker in not only the pregnancy-related disorders but also fetal diseases, for example, fetal congenital heart defects (CHD) [121].

## 4. Uterine MicroRNA

Uterus is a major female reproductive organ where fetus develops during gestation. There is no doubt that the miRNAs expressed in this organ are important for a healthy pregnancy. First of all, global effect of the uterine miRNAs in pregnancy was evaluated by knockdown of Dicer. In human endometrial stromal cells (hESCs), knockdown of Dicer caused decrease in decidualization marker and altered organization of actin filaments [122]. Although knockdown of Dicer only had a minor effect on the decidualization, 43 miRNAs were identified to be differentially expressed in decidualized hESCs compared to nondecidualized hESCs. Conditional knockout of Dicer in mouse uterine epithelium and stroma led to female sterility due to a small and significantly defective uterus [123]. Moreover, uterine miRNA expression profiles in the embryo implantation, term labor, and delivery were intensively analyzed. Hu and colleagues identified 8 differentially expressed miRNAs in the mouse uterus between implantation sites and interimplantation sites [124]. Furthermore, a group of 62 miRNAs were found to be dysregulated in the uterus between activation and delayed implantation [42]. Moreover, the expression of 226 miRNAs in human uterine cervix has been characterized and three miRNAs, miR-223, miR-34b, and miR-34c, were overexpressed in patients with spontaneous term labor [89].

### 4.1. Uterine MicroRNA Regulates Pregnancy Process

The function of individual uterine miRNA in pregnancy was revealed only partially. Early report in 2007 demonstrated that two uterine miRNAs, mmu-miR-101a and mmu-miR-199a^*∗*^, posttranscriptionally regulate a gene critical for implantation [91]. Three years later, another well-established cancer-associated miRNA, let-7a, was examined for the temporal and spatial expression in the uterus where the induction of let-7a was observed on the embryo invasion process [88]. Another member of let-7 family, let-7b, was found to be associated with preimplantation stages in epithelial cells as well as in uterus to inhibit uterine stromal cell proliferation [87]. Moreover, a microarray study identified 49 differentially expressed miRNAs between the noninduced endometrial stromal cells (ESCs) and induced ESCs [95]. One of these miRNAs, miR-222, regulates ESCs differentiation by direct targeting of cyclin-dependent kinase.

In another study, miRNAs were demonstrated to be important regulators of progesterone/estrogen signaling pathways. A cancer-associated miRNA family, miR-200, was implicated in uterus of both human and mouse preterm labor and served as progesterone/progesterone receptor- (PR-) mediated regulators [93]. Follow-up study discovered that miR-200a also directly targets STAT5b, a transcriptional repressor of the progesterone-metabolizing enzyme, and facilitates the progress to labor [94]. Another miRNA cluster, miR-199a/miR-214, was significantly decreased in laboring myometrium of pregnant mice and human and in a preterm labor mouse model [92]. Additionally, in rhesus monkeys, a set of endometrial miRNAs were differentially expressed during secretory phases and at least three miRNAs, miR-96, miR-375, and miR-219-5p, were the direct regulator of progesterone receptor [90]. On the contrary, progesterone or estrogen stimulation alters expression of uterine miRNA. In assisted reproduction cycles, the expression of miRNAs in the endometrium was differentially regulated with 3–5 days of progesterone treatment after retrieval (Zhao et al., 2012).

## 5. Maternal-Fetal MicroRNA Communication

Successful establishment of pregnancy requires coordination and interactions of maternal and fetal genes, proteins, and essential nutrients [125]. Growing evidence showed that inherited maternal miRNAs in the fetus are essential for fetal development. In mouse, the loss of maternal inheritance of miRNAs by specific deletion of Dicer in maturing oocyte caused the failure of the first cell division (Tang et al., 2007). Perturbing of the maternally inherited small nucleolar RNA (snoRNAs) and miRNAs resulted in embryonic lethality and developmental abnormalities of both embryo and placenta in mouse [126]. Maternal environmental and physiological factors can directly affect fetal miRNA expression, meaning that dysregulation of miRNA expression may lead to the fetal defects or even lifelong consequences. Maternal cigarette smoking during pregnancy downregulates cell growth and expression of developmental related miRNAs, miR-16, miR-21, and miR-146a, in the placenta [39]. Maternal low protein diet in mice altered the expression of mmu-miR-27a, mmu-miR-27b, and mmu-miR-330 which regulate brain renin-angiotensin system in the fetal offspring (Goyal et al., 2010). Moreover, maternal undernutrition amended the expression of fetal aortic miRNAs, which target the extracellular matrix remodeling and angiogenic factors in rat [15].

Moreover, fetal DNA and RNA, including miRNA, are presented in the maternal circulation and serve as important diagnostic tool for healthy pregnancy and fetal disease [127]. Previously, these fetal DNAs and RNAs were thought to be byproducts of fetal cell debris. However a recent study revealed that these small molecules have biological implications. MiRNAs released from cell surface and carried by exosome and exocytosis may act as mediators of cell-to-cell communication (Miura et al., 2010). These miRNA-containing exosomes can effectively communicate between different cell types and tissues under pathophysiological conditions and are actively involved in various cellular activities [128, 129].

**Figure 3:**
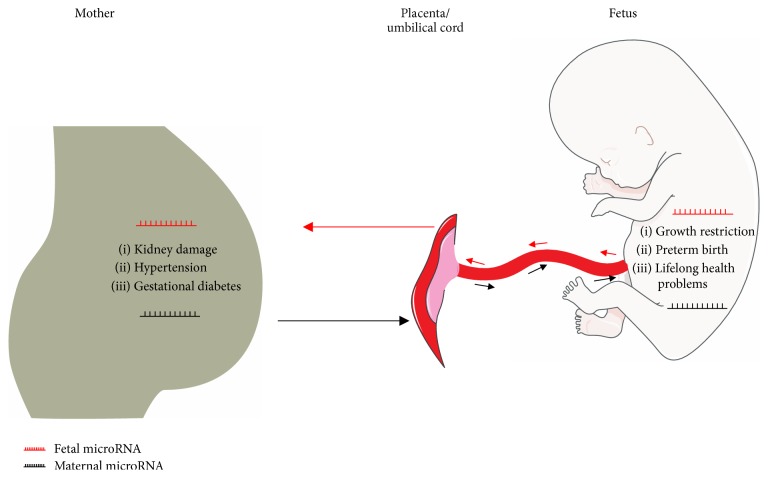
The mutual communication of miRNAs between fetus and mother in the pregnancy may link to most pregnancy-associated maternal and fetal disorders. The aberrant exchange of fetal and maternal miRNAs during pregnancy may even lead to lifelong problems in the fetus and mother.

Unfortunately, there are no reports on fetomaternal interaction of miRNAs. However, the possible regulatory roles of maternal miRNAs in fetal development and maternal pregnancy-associated disorders have been proposed [130]. It is evident that some pregnancy-associated maternal disorders, such as preeclamptic-related renal disease and pregnancy-induced hypertension, relieve their symptoms after parturition. Therefore, the fetal-origin substance is believed to be the root of these disorders [131]. These substances were initially proposed to be cells and DNA [132]. However, they are recently proved to be fetal RNAs, including mRNA and miRNA [86, 133, 134]. Despite the lack of functional characterization, the potential regulatory role of these fetal-maternal trafficking miRNAs is widely accepted. Essentially, the ectopic expression of these miRNAs may be linked to most pregnancy-associated maternal and fetal disorders (Figure 3).

## 6. Conclusions and Future Perspectives

Pregnancy is a complex and precisely regulated process. Loss of the balance between multiple physiological factors, such as oxygen concentration and inflammation, causes disorders or complications. For instance, the chronic hypoxia condition in the pathological placenta damages endothelium, leading to myocardial and microvascular dysfunctions, which is believed to be an initiating event of preeclampsia and IUGR [135, 136]. MiRNA as a “fine-tuner” is the best candidate for this kind of precise regulation. Indeed, the microarray data using placental and uterine tissue from different stages of pregnancy or pregnancy with complications suggested various miRNA expression patterns. The ability to suppress rather than degrade target mRNA makes miRNAs an effective tool for spatial and temporal regulation of gene expression in pregnancy and fetal development. Furthermore, the wide target range of 60% mammalian mRNAs ensures the importance of miRNA in the regulation of most physiological and pathological processes (Friedman et al., 2009). Particularly, in the regulation of pregnancy, miRNAs are shown to be actively involved in TGF and progesterone/estrogen signaling pathways, immune tolerance, inflammation, hypoxia, fetus development, preeclampsia, and IUGR. Thus, we can speculate that many other miRNAs which relate to the fundamental cellular and tissue activities will also play roles in the regulation of pregnancy.

The function of these pregnancy-related miRNAs is still elusive. Current data of miRNA expression patterns, even in the similar experimental and sampling conditions, can be significantly different. Despite the controversy in the literature, current studies still confirm that miRNAs are involved in many aspects of pregnancy and its disorders. MiRNAs were demonstrated to contribute to the immune tolerance in pregnancy, mesenchymal cell differentiation, and angiogenesis. Several miRNAs are shown to be the regulators in trophoblast cells proliferation, migration, and invasion, and the dysregulated expression of these miRNAs was demonstrated in the tissues of preeclampsia or IUGR. However, there are many other aspects that are largely limited, such as fertilization, implantation, cleavage, decidualization, gestation, and parturition. Placenta is the most studied tissue type and very important fetal-maternal organ. Unfortunately, uterus or other pregnancy-related organs have been less extensively studied but are no less important. Collectively, current studies on pregnancy-associated miRNAs are primitive with the main focus on trophoblast and mesenchymal stem cells, while stromal and endothelial cells that are essential to placental vasculature and angiogenesis have been ignored.

Another important but less studied subject is the circulating miRNA. Successful pregnancy requires the interaction and communication between maternal and fetal factors. Current studies are mainly focusing on the biomarker potential of these miRNAs and the regulatory and biological functions of these miRNAs in pregnancy and its disorders have not being proposed [134]. Considering the fact that many pregnancy-associated disorders disappear after giving birth, exchange of maternal-fetal substance, especially miRNAs, could be the key to understand the pregnancy disorders.

Although miRNAs are proposed to be important diagnostic markers and therapeutic targets, current applications of these miRNAs are very limited. A recent study suggested that serum miRNA assessment in the first trimester of pregnancy showed no predictive value in early onset preeclampsia [137]. The main reason for this is the complexity of miRNA biology, leading to contradiction in the literature. A study compared eight previously published array data sets with their own microarray data and revealed a total of 138 differentially expressed miRNAs in preeclamptic placenta. However, only 14% of these miRNAs were seen in more than one study and the results agreed in the direction of change [138]. In addition, miRNA expression profiling in maternal peripheral blood and placental tissue within the same group of patients is completely altered and the expression profile changes in different gestational ages [118, 139]. Such contradiction may arise due to the different time point or position of sample collection and even different human population. Some miRNAs were suggested to be only strongly expressed during narrow time windows [140], while others were expressed oppositely in different ethnic groups [141]. Taken together, miRNA studies in pregnancy and its associated disorders are still in the primary stage. Nevertheless, the potential application of pregnancy-associated miRNAs in the medical diagnosis and treatment of pregnancy-associated disorders is promising. Future studies are guaranteed to enrich our understanding of pregnancy-associated disorders and provide novel diagnostic and therapeutic targets.

## Figures and Tables

**Table 1:** A brief list of pregnancy-related microRNAs.

MicroRNA name	Function relates to pregnancy	Reference
Placenta-specific microRNA		

miR-23a, miR-136, and miR-141	Enriched in placenta; trophoblast cell proliferation; target pleiomorphic adenoma gene 1 (PLAG1)	[23–25]

C14MC cluster	Abundant in developing embryo and placenta	[26]

C19MC cluster (miR-516, 517, 518, 519, etc.)	Expression restricted in reproductive system and placenta; trophoblast cell invasion, migration; implicated in complete hydatidiform moles (CHM), fetal growth restriction (FGR)	[27–32]

miR-371, miR-372, and miR-373	Predominantly expressed in placenta	[33]

miR-675	Inhibits embryonic and extraembryonic cell lines proliferation	[34]

miR-1302 family	Found in mammal placenta	[35]

Placenta-associated microRNA		

Let-7	Regulates NF-*κ*B pathway and IL-6	[36]

miR-15a, miR-15b	Regulates angiogenesis and is increased in preeclampsia; differentially expressed in preterm birth placenta	[37, 38]

miR-16, miR-21, and miR-146a	Downregulated in response to smoke exposure, angiogenesis-associated, and decreased in the small for gestation (SGA) placentas	[39, 40]

miR-17∼92 cluster	Regulate syncytiotrophoblast differentiation	[41]

miR-17, miR-27, and miR-92	Differentially expressed in gestation age; downregulated in early pregnancy loss placenta	[42, 43]

miR-17, miR-20a, and miR-20b	Increased in preeclampsia and regulate hydrogen sulphide (H_2_S)	[44, 45]

miR-18a	Inhibits invasion and promotes apoptosis of trophoblast cells	[46]

miR-19b	Downregulated in early pregnancy loss placenta	[43]

miR-27a, miR-199b, and miR-429	Regulate renin-angiotensin system to help placenta adapt to hypoxia	[47]

miR-29b	Regulates apoptosis, invasion, and angiogenesis of trophoblast cells	[48]

miR-30a	Attenuates mesenchymal stem cells (MSCs) mediated immune response in preeclampsia	[44]

miR-34a	It is decreased in placenta accreta patients; and suppression of miR-34a increased trophoblast invasion	[49]

miR-93, miR-205, miR-224, miR-335, miR-424, miR-451, miR-491	Differentially expressed in primary trophoblasts exposed to hypoxia and downregulate eNOS (miR-335)	[50, 51]

miR-101	Regulates apoptosis of trophoblast cells	[52]

miR-126	Proangiogenic factor and decreased in preeclampsia	[53]

miR-133a	Overexpressed in recurrent spontaneous abortion (RSA) and downregulates human leukocyte antigen (HLA)-G	[54]

miR-137	Affects proliferation and migration of placenta trophoblast cells	[55]

miR-148/152 family	Inhibits IL-12, IL-6, and TNF-*α* and mediates immune tolerance; regulates HLA-G	[56, 57]

miR-155	Regulates trophoblast function, including proliferation, migration, invasion, and differentiation; downregulates angiogenic factors and implicated in preeclampsia; targets angiotensin II type 1 receptor; modulates eNOS expression	[58–62]

miR-181	Regulates TGF-*β* pathway and IL-6; attenuates the immunosuppressive properties in placenta; inhibits proliferation and immunosuppressive properties of MSCs; is differentially expressed in preterm birth placenta	[37, 63]

miR-195	Altered expression in preeclampsia; affects trophoblast cell invasion	[31, 64–67]

miR-210	Increased expression in response to the hypoxic placenta; regulates trophoblast cell migration and invasion; modulates mitochondrial respiration in placenta; targets critical steroidogenetic enzyme; modulates inflammation-related pathway, potassium channel modulatory factor, and thrombospondin; increased in FGR; differentially expressed in preterm birth placenta	[37, 68–75]

miR-325	Elevated in preeclampsia and correlates with blood pressure	[76]

miR-376c	Promotes trophoblast cell proliferation and invasion	[77]

miR-378a-5p	Promotes trophoblast cell survival, migration, and invasion and contributes to preeclampsia	[78]

miR-424	Regulates trophoblast differentiation	[79]

miR-494	Inhibits MSCs proliferation and angiogenesis	[80]

miR-675	Regulates placental trophoblast cell proliferation	[81]

Placenta-derived circulating microRNA		

C14MC cluster	Identified in the circulation of pregnancy women	[82]

C19MC cluster	Identified in exosome and released from human primary trophoblast; upregulated in preeclamptic plasma; increased at early gestational age in circulation	[83–85]

miR-127, miR-134, and miR-498 cluster	Uniquely correlated in the maternal and fetal circulation	[82]

miR-135b, miR-141, miR-149, and miR-299-5p	Detected in maternal plasma during pregnancy and decreased in postdelivery plasma	[86]

Uterine microRNA		

Let-7a, let-7b	Induction on the process of embryo invasion during implantation; gradually increased in uteri to inhibit uterine stromal cell proliferation	[87, 88]

miR-34b, miR-34c, and miR-223	Overexpressed in spontaneous term labor	[89]

miR-96, miR-219-5p, and miR-375	Differentially expressed during prereceptive and receptive phase; regulator of progesterone receptor	[90]

mmu-miR-101a and mmu-miR-199a^*∗*^	Regulate critical gene for implantation	[91]

miR-199a/miR-214	Decreased in laboring myometrium and in an inflammatory preterm labor mouse model	[92]

miR-200	Implicated in uterus of preterm labor; involved in progesterone/progesterone receptor pathway	[93, 94]

miR-222	Regulates endometrial stromal cells (ESCs) differentiation	[95]
